# Competition for Chaperones: A Trade-Off Between Thermotolerance and Antiviral Immunity in Plants

**DOI:** 10.3390/cimb47110957

**Published:** 2025-11-18

**Authors:** Almas Madirov, Nurgul Iksat, Zhibek Turarbekova, Bakhytbek Abzhalelov, Zhaksylyk Masalimov

**Affiliations:** 1 Scientific Laboratory for Plant Biotechnology Named After Rustem Omarov, L. N. Gumilyov Eurasian National University, Astana 10000, Kazakhstan; almasmadirov@gmail.com (A.M.);; 2 Institute of Engineering and Technology, Korkyt Ata University, Kyzylorda 120014, Kazakhstan

**Keywords:** plant immunity, heat stress, combined stress, viral infection, HSP90, HSP70, molecular chaperones, NLR receptors, proteostasis, trade-off, climate change, crop resilience

## Abstract

Molecular chaperones HSP70 and HSP90 represent a critical, yet conflict-ridden, node in plant physiology, particularly under the dual impact of heat stress and viral infection. As key components of both thermotolerance (maintaining proteostasis) and innate immunity (stabilization of NLR receptors), they are simultaneously exploited by viruses to facilitate their own life cycle. This review critically analyzes this “trilemma,” focusing on the hypothesis of competition for a limited chaperone pool. We present mechanistic insights indicating that during heat stress, cellular priority shifts towards maintaining global proteostasis, thereby diverting chaperones from immune functions. This resource-based competition mechanism potentially explains the collapse of ETI-immunity, as NLR receptors, deprived of support from the HSP90-SGT1-RAR1 complex, are destabilized and targeted for degradation. We also integrate adjacent signaling pathways into this model, including hormonal cross-talk (SA, JA) and autophagy. Understanding this trade-off opens new perspectives for molecular breeding and the biotechnological engineering of stress-resilient crop varieties.

## 1. Introduction

In the context of global climate change, plants are increasingly confronted with the simultaneous impact of multiple stressors [1,2]. The convergence of abiotic stress, such as anomalous heat, and biotic stress from viral infection poses a particular threat to agricultural crops and natural ecosystems [1,3]. The outcome of this complex interaction is determined at the molecular level, where cellular systems must respond concurrently to fundamentally different threats [4,5].

At the heart of these processes are molecular chaperones, particularly the Heat Shock Protein 70 (HSP70) and Heat Shock Protein 90 (HSP90) families [6]. These ubiquitous components of the protein homeostasis (proteostasis) network play a paradoxical role: they are key elements of both acquired thermotolerance and innate immunity [7]. However, lacking their own chaperones, viruses have evolved to exploit this same cellular machinery to facilitate their life cycle. This creates a fundamental conflict of interest in which HSPs become a limited resource, contested by the cell’s survival systems, its immune defenses, and the viral pathogen [8].

To unravel this conflict, we rely in this review on the “competition for a limited resource” hypothesis. Notably, while this hypothesis is widely supported by genetic and physiological data, it largely remains a conceptual framework; direct biochemical evidence for competitive binding in planta under stress conditions is still accumulating. This work aims to systematize existing evidence, beginning with the architecture of the chaperone machinery and its dual (pro-and antiviral) roles. We then analyze the destabilizing impact of heat stress on the components of this system and, finally, present an integrative model explaining the collapse of immunity, as well as consider emerging data on adjacent pathways (autophagy, hormones) and applied biotechnological perspectives.

## 2. The HSP70 and HSP90 Machinery in Plants: Architecture, Mechanisms, and Regulation

The molecular chaperones HSP70 and HSP90 are fundamental components of the cellular proteostasis network [9]. In plants, as sessile organisms forced to constantly adapt to changing environmental conditions, this machinery has attained remarkable complexity [10]. It not only ensures the proper folding and functional activity of proteins under normal conditions but also serves as a central hub in the response to biotic and abiotic stresses. Understanding the architecture, mechanisms, and regulation of these chaperones is key to deciphering plant survival strategies.

The functional diversity of chaperones is enabled by the existence of numerous isoforms localized in different cellular compartments. The HSP70 family, one of the most evolutionarily conserved, includes both stress-inducible proteins (HSP70) and constitutively expressed forms (HSC70) [11]. For example, the Arabidopsis thaliana genome encodes 18 HSP70 and 7 HSP90 genes, but in key agricultural crops, this number is often significantly higher, reflecting their complex genomes and adaptive needs. The rice (Oryza sativa) genome encodes 30 HSP70 and 9 HSP90 genes; 22 ZmHsp70 and at least 2 ZmHsp90 genes have been identified in maize (Zea mays); and in common wheat (Triticum aestivum), this family is exceptionally expanded, comprising 113 HSP70 genes [12,13,14,15,16,17]. The functional diversification of these isoforms is frequently investigated using transient expression systems in tobacco or Arabidopsis protoplasts, as well as through the analysis of T-DNA knockout mutants, to elucidate isoform-specific roles in development, thermotolerance, or immunity [18,19,20,21,22]. This genetic diversity allows chaperones to be present and perform specific tasks in the cytosol, nucleus, endoplasmic reticulum (ER), mitochondria, and chloroplasts [23]. Their precise localization is determined by conserved signaling motifs, such as the C-terminal EEVD sequence for cytosolic forms or the HDEL motif for ER-resident proteins, known as BiP [7,24].

The function of these chaperones is based on dynamic, ATP-dependent cycles of client protein binding and release [25,26]. In the HSP70 cycle, co-chaperones known as J-domain proteins (JDPs or HSP40s) play a crucial role [27,28]. They recognize and deliver a client protein to HSP70 and stimulate ATP hydrolysis, which shifts the chaperone into a high-affinity conformation, ensuring substrate capture. The subsequent exchange of ADP for ATP, catalyzed by nucleotide exchange factors (NEFs), returns HSP70 to its low-affinity state, releasing the mature client [29]. The vast diversity of JDPs in plants enables the HSP70 system to recognize a wide spectrum of substrates and direct its activity toward specific cellular processes [28].

The maturation of many complex proteins, especially those involved in signal transduction, requires the sequential action of the HSP70 and HSP90 cascade. The transfer of a client between them is coordinated by the co-chaperone HOP (HSP70-HSP90 Organizing Protein), which acts as a molecular bridge by simultaneously binding both chaperones to form a transient ternary complex [30]. This machinery is of particular importance in the plant innate immune system. HSP90, in cooperation with the co-chaperones SGT1 and RAR1, forms a complex that is critical for maintaining the stability and signaling competence of intracellular NLR (Nucleotide-binding Leucine-rich repeat) immune receptors, also known as R-proteins [7]. These receptors are inherently metastable and require constant “maintenance” from the chaperone complex to be maintained in an activation-competent state [31].

**Figure 1:**
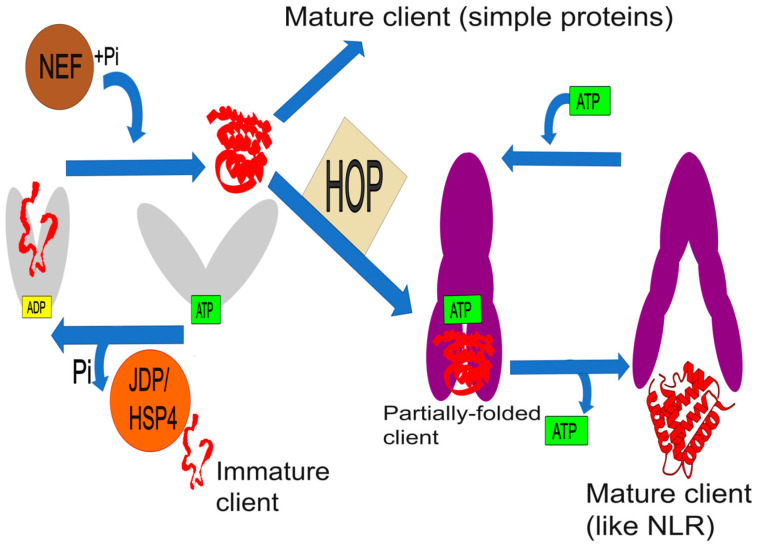
Model of the HSP70-HSP90 chaperone conveyor, illustrating the “triage” of client proteins. An Immature client enters the ATP-dependent HSP70 cycle, assisted by the JDP (HSP40) co-chaperone, while a NEF catalyzes release. This node acts as a “sorting center”: most ‘simple’ proteins fold completely and exit the HSP70 cycle as functional Mature clients. However, a more complex subclass of clients (such as NLR receptors), remaining in a Partially folded state, are transferred via the HOP co-chaperone to the specialized HSP90 machinery. The final ATP-dependent HSP90 cycle completes their maturation, releasing the Mature client (e.g., an NLR receptor). Adapted from Hervás and Oroz (2020) [38].

HSP expression is under the stringent control of Heat Shock Transcription Factors (HSFs), which are activated in response to proteotoxic stress [32]. The classic model of their activation is based on the “chaperone titration” principle [33]. Under normal conditions, HSFs are held inactive in a complex with HSP70/HSP90. During stress, chaperones are diverted to accumulated denatured proteins, releasing HSF monomers [34]. The liberated HSFs trimerize, translocate to the nucleus, and bind to heat shock elements (HSEs) in the promoters of *HSP* genes, triggering their robust expression. The HSF family in plants is particularly complex and hierarchically organized, with “master regulators” such as HSFA1 controlling the activity of secondary factors like HSFA2, ensuring a large-scale transcriptional response [35,36,37]. The architecture of this complex machinery, encompassing the HSP70 and HSP90 cycles, as well as its connectivity to immune system clients such as NLR receptors, is schematically depicted in Figure 1. This regulatory network integrates signals not only from heat stress but also from other stimuli, including reactive oxygen species and calcium, and can be specifically suppressed by viral proteins to overcome the host’s defensive barriers.

## 3. The Dual Role of Chaperones in Viral Infections

Having examined the complex architecture and regulatory mechanisms of the chaperone machinery, it is necessary to analyze its central yet paradoxical role during viral infection. Molecular chaperones HSP70 and HSP90 act in this arena as classic “double agents.” On one hand, they are indispensable components of the plant’s innate immunity; on the other, they are actively exploited by viruses, which, lacking their own chaperones, are entirely dependent on host cellular systems to complete their life cycle (Table 1).

### 3.1. Pro-Viral Functions: Exploitation of Chaperones for Viral Propagation

**Figure 2:**
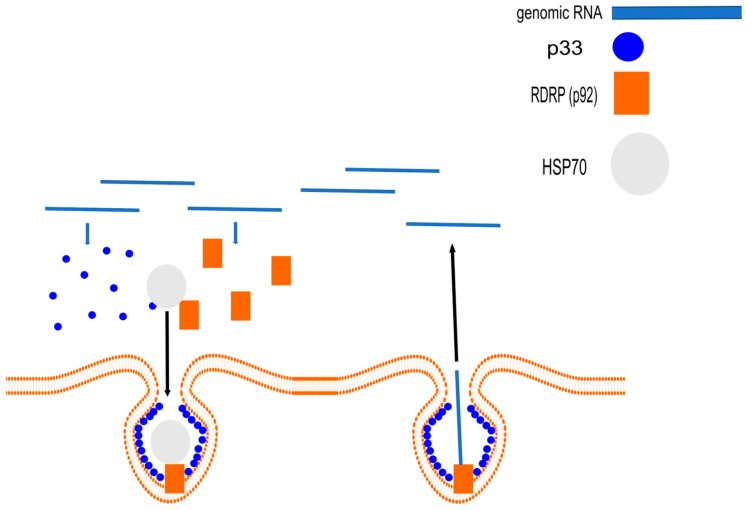
Mechanism of the pro-viral function of chaperones. The diagram illustrates how a virus hijacks the host chaperone HSP70 (gray circle) for the assembly of the Viral Replication Complex (VRC). Using Tomato bushy stunt virus (TBSV) as an example, HSP70 is required for the correct localization and insertion of viral replicase proteins (the auxiliary protein p33, blue circle, and the p92-RdRp, orange square) into organelle membranes (orange line). This forms protected vesicles (VRCs) that then import genomic RNA (blue line) for replication and export new copies. (Adapted from [39]).

To successfully colonize a host, viruses have evolved to recruit chaperones, especially HSP70, at nearly every stage of their development, from genome replication to the assembly and spread of viral particles These interactions can be broadly divided into two groups: (1) direct binding of chaperones to viral proteins (e.g., replicase or capsid proteins) to facilitate their folding, assembly, and transport; and (2) indirect manipulation, where chaperones modulate host cellular proteins (e.g., RNA silencing components) that the virus exploits for its benefit. One of the key pro-viral functions is the involvement of HSP70 in the formation and function of viral replication complexes (VRCs) [39,40,41]. Many RNA viruses create membrane-associated compartments where their genome replication occurs [50]. Cytosolic HSP70s are recruited for the correct localization and insertion of viral replicase proteins into organellar membranes, such as peroxisomes or the endoplasmic reticulum, as well as for the conformational activation of the RdRp. A classic example is Tomato bushy stunt virus (TBSV), whose replicase proteins remain inactive in the cytosol without the assistance of HSP70, completely blocking replication [39,40,41]. This mechanism of chaperone hijacking for VRC assembly is illustrated in Figure 2.

Similarly, in rice, the host HSP70 (OsHSP70) is required for Rice stripe virus (RSV) infection, as it interacts with the N-terminus of the viral [51]. In wheat, a positive role for HSP70 has also been demonstrated in the replication of Chinese wheat mosaic furovirus [52]. Replicase of Red clover necrotic mosaic virus (RCNMV) forms a multiprotein complex on the ER membrane that includes both HSP70 and HSP90 [42]. HSP70 appears to prevent the non-specific aggregation of the replicase protein, while HSP90 is required for a later step: ensuring the binding to a specific viral RNA element, which is critical for proper VRC assembly. Beyond replication, chaperones facilitate the folding, stabilization, and transport of viral components. HSP70 prevents the aggregation and degradation of newly synthesized viral proteins, the accumulation of which would otherwise induce proteotoxic stress in the cell. Chaperones are also necessary for the movement of viral proteins and particles within and between cells [53]. For instance, HSP70 participates in the nuclear import of the coat protein of Tomato yellow leaf curl virus (TYLCV), where viral factories are assembled [43]. Furthermore, HSP70 can facilitate the intercellular movement of viruses through plasmodesmata. Some viruses, such as those in the *Closterovirus* genus, even encode their own HSP70 homolog (HSP70h), which functions as a specialized movement protein to ensure virion transport from cell to cell [54]. Consequently, suppressing HSP70 activity, whether through genetic engineering or with inhibitors, effectively curtails infection by a broad spectrum of plant viruses, confirming the critical importance of this chaperone for pathogenesis.

### 3.2. Anti-Viral Functions: HSPs as Sentinels of Cellular Immunity

The most important anti-viral function of chaperones is the maintenance of effector-triggered immunity (ETI), which represents the most robust line of defense against pathogens [55,56]. ETI is initiated by intracellular NLR receptors, which are inherently metastable proteins [57,58]. HSP90, together with the co-chaperones SGT1 and RAR1, forms a dynamic complex that stabilizes NLR proteins, maintaining them in an inactive but activation-competent state [59,60,61]. Unlike in animals, plant NLR proteins typically recognize not the pathogenic molecules themselves but their activity, using a mechanism of indirect recognition. This principle is described by the “guard” hypothesis, where an NLR “guards” a target protein, and the “decoy” hypothesis, where a decoy protein attracts an effector [62]. The molecular trigger for NLR signaling involves conformational changes (ADP to ATP exchange) and subsequent oligomerization, leading to the activation of N-terminal domains (TIR or CC) that initiate programmed cell death [63,64]. Coordinated localization between the nucleus and cytoplasm is critical for their function; for example, the nuclear pool of the TIR-type receptor RPS4 is essential, whereas the CC-type receptor Rx is activated in the cytoplasm [62,65].

Disruption of the chaperone complex function leads to the degradation of NLR receptors and a loss of pathogen resistance. For instance, tobacco’s resistance to Tobacco mosaic virus (TMV), mediated by the N protein, is entirely dependent on the functionality of this complex [44,47]. Cytosolic HSP70s are also involved in this intricate regulatory network, cooperating with the HSP90-SGT1-RAR1 complex to modulate immune responses. However, the complex also appears to participate in the negative regulation and turnover of NLR proteins, likely to prevent autoimmune reactions or inappropriate activation. For example, in sgt1b mutants, some NLR proteins accumulate to abnormally high levels, indicating a role for SGT1 in their degradation [65,66]. This reframes the complex’s function from simple stabilization to active homeostatic control. It acts as a quality control hub that balances the need for a ready pool of receptors against the danger of false alarms.

Beyond ETI, chaperones contribute to other layers of defense. They play an important role in pattern-triggered immunity (PTI) by ensuring the quality control of membrane-bound pattern recognition receptors (PRRs) that recognize conserved pathogen-associated molecular patterns [67]. The ER-resident HSP70, known as BiP, is essential for the proper maturation and transport of these receptors to the cell surface [68]. Furthermore, HSP90 is a key participant in RNA silencing, a primary antiviral defense mechanism in plants [48,49]. It interacts with Argonaute (AGO) proteins and is necessary for the efficient loading of small RNAs into the RNA-induced silencing complex (RISC), which recognizes and destroys viral RNA. Thus, chaperones act as multifunctional regulators that not only maintain the stability of key immune receptors but also ensure the functionality of other critical defense systems.

Thus, Section 3.1 and Section 3.2 depict a fundamental conflict. On one hand, the virus is evolutionarily “programmed” to hijack HSP70 and HSP90 for the assembly of replication complexes and transport. 5 On the other hand, the host immune system, particularly ETI, is absolutely dependent on the same HSP90-SGT1-RAR1 complex to maintain the “readiness” of its NLR receptors. 5 Under normal conditions, the cell apparently maintains a sufficient chaperone pool for both tasks. However, this fragile equilibrium is completely disrupted by the intervention of a third, dominant factor: heat stress.

## 4. The Impact of Temperature Stress on System Components

Heat stress acts as a powerful modulator that alters the rules of engagement in the plant-virus system. It directly affects each of the three key components: the host’s chaperone machinery, the virus’s life cycle, and the efficacy of the plant’s immune system. The outcome of the confrontation under combined stress is determined by this complex and often non-linear influence of temperature.

### 4.1. HSPs and Thermotolerance: Maintaining Cellular Proteostasis

The primary function of heat shock proteins at elevated temperatures is to maintain cellular proteostasis. Heat stress causes the denaturation and aggregation of proteins, and chaperones like HSP70 and HSP90 shoulder the primary burden of refolding, stabilizing, and, in cases of irreversible damage, targeting them for degradation via the ubiquitin-proteasome system [69]. The massive induction of HSP genes, known as the heat shock response (HSR), is governed by HSF transcription factors and is the central element of acquired thermotolerance [70]. While numerous chaperones participate in this process, the key, indispensable isoform for acquired thermotolerance in Arabidopsis is the cytosolic chaperone HSP101 [71]. Hot1 mutants, lacking HSP101, are unable to survive lethal temperatures despite prior acclimation, demonstrating its central role in refolding aggregates [72,73]. 21 Small HSPs (sHSPs), such as the HSP20 family, also play a crucial role, acting as a “first line of defense” by binding denatured proteins prior to their refolding [74]. This mechanism allows a plant that has survived a moderate heat stress to endure subsequent, potentially lethal temperatures. Thus, HSPs form the cell’s first line of defense, protecting its proteome from thermal damage and ensuring the organism’s survival.

### 4.2. Temperature Effects on the Viral Lifecycle

Temperature has a dual effect on the replication and spread of viruses, which depends on the specific virus–host pair and the temperature regime [75]. On one hand, elevated temperature can have a pro-viral effect [76,77]. This is often linked to the suppression of host immune responses, as discussed below. Furthermore, heat-induced HSPs are actively recruited by viruses to facilitate replication and transport. For example, in tobacco plants infected with Potato virus Y (PVY), heat shock accelerated the systemic spread of the pathogen, which correlated with high levels of HSP70 and HSP90 [78,79]. This effect is also strongly modulated by virus type (e.g., rapidly replicating RNA viruses may more quickly exploit heat-induced immune suppression) and by host developmental stage, as young, actively growing tissues are often more susceptible to both stress and the virus [80,81].

On the other hand, temperature can also have an anti-viral effect. In some cases, elevated temperatures directly inhibit the activity of viral replicases, as has been shown for TBSV [5]. Additionally, temperature can enhance defense responses, particularly RNA silencing, leading to a reduction in viral load. This principle underlies thermotherapy, a method used to eliminate viruses from plant material through prolonged exposure to elevated temperatures. It is also important to note that temperature critically affects the life cycle and behavior of insect vectors, modulating viral accumulation within them and the efficiency of transmission, which directly impacts the epidemiology of viral diseases [82].

### 4.3. Temperature Sensitivity of Plant Immunity

Plant defense systems are highly sensitive to temperature fluctuations, and their suppression under heat stress is often the primary cause of increased susceptibility to pathogens [83,84]. This applies to the two main pillars of antiviral defense: effector-triggered immunity and RNA silencing.

The primary reason for the temperature sensitivity of ETI is the instability of NLR receptors [85,86]. As previously shown, the HSP90-SGT1-RAR1 chaperone complex is required to maintain their functional state. Elevated temperature creates a situation where chaperones, primarily HSP90, are massively diverted to refold other cellular proteins damaged by heat. This leads to the “unmasking” of NLR proteins, their destabilization, and subsequent degradation, ultimately compromising the entire ETI system. A classic example is the suppression of N protein-mediated resistance to TMV in tobacco at temperatures above 28 °C [87]. In another example, potato resistance to Potato virus X, mediated by the Rx protein, is also compromised at high temperatures [88]. In Arabidopsis, autoimmune phenotypes caused by the hyperactive NLR mutant SNC1 are suppressed at elevated temperatures, indicating protein destabilization [89]. This genetic evidence serves as strong indirect proof that the HSP90 complex is the ‘Achilles’ heel’ of ETI under heat stress.

The effect of temperature on RNA silencing is more complex. As a rule, low temperatures inhibit this defense mechanism by reducing the accumulation of small interfering RNAs (siRNAs), making plants more vulnerable [88]. The impact of high temperatures is dual: in some cases, they can enhance RNA silencing, promoting recovery from infection, while in others, they can weaken it, especially in synergy with viral suppressors of silencing [89,90]. Thus, temperature acts as a critical switch that can either bolster or completely dismantle the plant’s key defensive barriers, determining the outcome of its interaction with a virus.

Although the thermosensitivity of NLR proteins is a general paradigm, it is important to note that this is not a universal rule. Exceptions exist, indicating a diversity of thermosensitivity mechanisms. For instance, while pepper resistance to Tobacco mild green mosaic virus mediated by L1–L2 is suppressed at high temperatures, L1a-mediated resistance remains stable [91,92]. This suggests that not all NLR proteins share the same dependency on the HSP90 complex, or that some possess intrinsic thermostability.

## 5. Beyond the Chaperone

### 5.1. Hormonal Crosstalk: SA, JA, and Thermotolerance

The conflict between thermotolerance and immunity is not restricted to chaperones; it is deeply embedded within the hormonal signaling network. A key antagonism exists between salicylic acid (SA), essential for defense against biotrophic and hemibiotrophic pathogens (including viruses), and jasmonic acid (JA), which mediates defense against necrotrophs and herbivores [93]. In response to heat stress, both the salicylic and jasmonic signaling pathways are activated [94]. However, the mutual negative regulation on one hand, and the positive regulation by stress on the other, can disrupt resource allocation [95,96]. Abscisic acid (ABA), which is also activated in response to stress, further complicates this interplay, as it acts jointly with JA to suppress SA [93]. This heat-induced shift from SA towards JA/ABA represents another trade-off, whereby the plant reallocates resources from antiviral defense (SA) to JA-mediated general stress resilience.

### 5.2. Autophagy: A Critical Hub for Proteostasis and Virophagy

Autophagy, the process of cellular “self-digestion,” constitutes the second pillar of proteostasis alongside chaperones [97]. It is intimately linked with both types of stress. First, autophagy plays a pivotal role in antiviral defense [98]. A mechanism known as “virophagy,” or selective autophagy, targets viral components and virions for degradation in the vacuole. For example, autophagy has been shown to target viral proteins, such as βC1 in geminiviruses or HCpro in potyviruses, thereby restricting infection [99]. Second, autophagy is critical for thermotolerance, clearing irreversibly damaged protein aggregates that the chaperone system failed to resolve [100]. However, during recovery from heat stress, autophagy is also involved in “resetting” the cellular state by removing protective macromolecules, including chaperones themselves, thereby allowing the cell to return to normal growth [101,102]. Thus, under combined stress, another conflict emerges: should autophagy attack the virus or clear the cell of thermal aggregates?

## 6. An Integrative Model: HSPs at the Nexus of Heat and Viral Stress

When viral infection and heat stress occur simultaneously, the cellular machinery finds itself at the epicenter of a conflict of interest, with molecular chaperones as the focal point. We have previously considered their separate roles, but it is under combined stress that their multitasking becomes a fundamental trade-off between survival and defense. A comparison of these conflicting priorities and their outcomes for the cell is presented in Table 2.

**Figure 3:**
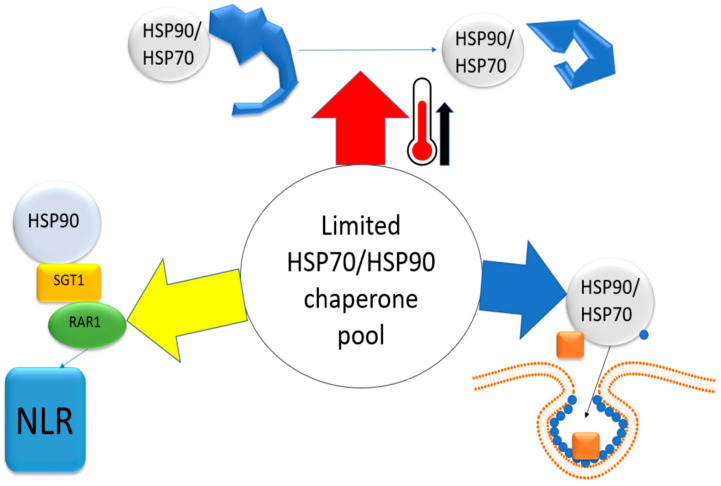
A conceptual model of the competition for the limited chaperone pool (HSP70/HSP90) under combined heat stress and viral infection. The central, limited pool of molecular chaperones is a resource simultaneously required by three key processes: (**Left**) Immunity Maintenance: The HSP90-SGT1-RAR1 chaperone complex is essential for stabilizing intracellular NLR immune receptors, keeping them in an activation-competent state. (**Right**) Viral Lifecycle Support: Viruses actively exploit host chaperones, primarily HSP70 and HSP90, for the correct assembly and function of viral replication complexes (VRCs) within cellular membrane structures. (**Top**) Thermotolerance and Proteostasis: During heat stress, HSP70 and HSP90 perform the vital function of refolding and stabilizing denatured cellular proteins to maintain proteome integrity.

HSPs must simultaneously perform three mutually exclusive tasks: rescue cellular proteins from heat-induced denaturation (thermotolerance), maintain the functionality of immune receptors (antiviral defense), and, against their will, assist the virus in its replication and assembly (pro-viral function). This fundamental conflict for a limited chaperone resource is illustrated in Figure 3.

### 6.1. Competition for Chaperones and the Immunity-Thermotolerance Trade-Off

The central hypothesis explaining the outcome of this confrontation is based on competition for a limited pool of chaperones. During heat stress, the cell initiates a massive expression of HSP genes to cope with the vast number of denatured and misfolded proteins. This creates a colossal demand for chaperone activity. In this situation, the cell is forced to allocate resources, and the priority becomes survival-that is, maintaining global proteostasis to prevent the collapse of cellular functions. An inevitable trade-off arises: resources directed toward ensuring thermotolerance are diverted from other systems, primarily from immunity.

### 6.2. The Molecular Mechanism of Immunity Collapse: A “Resource Depletion” Hypothesis

The temperature-dependent collapse of immunity, particularly ETI, can be explained by the “resource depletion” hypothesis. As established, NLR immune receptors are metastable proteins that require constant “maintenance” by the HSP90 complex to preserve their functional conformation. During heat stress, the vast majority of free and newly synthesized HSP90 is immediately diverted to the higher-priority task of refolding vital cellular proteins. Admittedly, this model, while logically sound and supported by genetic data (e.g., the thermosensitivity of SNC1 mutants, relies heavily on a conceptual framework rather than on direct biochemical measurements of client competition in planta. Quantifying the partitioning of the HSP90 pool between NLR receptors and denatured proteins during heat shock remains a key challenge for future research. As a result, NLR receptors are deprived of their necessary chaperone support, become destabilized, and are likely recognized by the quality control system as defective, after which they are targeted for degradation. This very mechanism underlies the classic example of the collapse of N-mediated resistance to TMV in tobacco above 28 °C. The immune system is effectively dismantled due to a shortage of resources that have been reallocated to combat overheating.

### 6.3. The Influence of Stress Dynamics: Acclimation Versus Shock

The outcome of this conflict depends heavily on the dynamics of the temperature exposure. A gradual and moderate increase in temperature can lead to acclimation: the cell has time to accumulate an elevated basal level of HSPs, which serves as a buffer, increasing resilience to subsequent stress events. In contrast, a sudden and severe heat shock causes immediate and massive protein damage, leading to an acute depletion of the free chaperone pool. It is under such shock conditions that the most rapid and complete collapse of NLR-mediated immunity occurs, due to an acute shortage of available HSP90. Thus, not only the magnitude but also the rate of stress onset determines whether the plant can adapt or if its defense systems will be compromised.

## 7. Conclusions and Future Perspectives

In this review, we have analyzed the complex and multifaceted role of the molecular chaperones HSP70 and HSP90 at the intersection of heat stress and viral infection in plants. The data presented compellingly demonstrate that chaperones are not merely passive executors but a central hub where stress signals are integrated and the conflict between survival and defense is resolved. The competition for a limited chaperone resource among the processes of proteome maintenance, immune receptor stabilization, and the demands of viral pathogenesis is the key mechanism determining the outcome of the interaction. By diverting the main pool of HSPs to combat the denaturation of cellular proteins, heat stress creates a “window of vulnerability,” leading to the destabilization and degradation of NLR receptors and, consequently, to the collapse of genetically determined resistance.

Despite significant progress, several fundamental questions remain open, defining future research directions. First, it is necessary to decipher the functional specificity of the numerous HSP isoforms. Is there a division of labor, where some isoforms are primarily responsible for immunity while others handle thermotolerance or become targets for viruses? Second, it is critically important to understand the role of co-chaperones and the stoichiometry of chaperone complexes in determining client priority under combined stress. Third, the trade-off hypothesis itself requires quantitative assessment: what fraction of the chaperone pool is diverted to each stress, and what is the threshold beyond which the immune system collapses? Finally, a promising avenue is the exploration of genetic engineering-is it possible to “uncouple” the pro-viral functions of HSPs from their vital cellular roles by modifying co-chaperones or HSF regulators? Answering these questions will not only deepen our fundamental knowledge of plant stress physiology but also pave new ways for developing crop varieties resilient to the challenges of a changing climate.

Understanding the trade-off between thermotolerance and immunity opens direct avenues for the biotechnological improvement of crops. One of the most promising strategies is the engineering of thermostable NLR receptors. Another approach is “uncoupling” the pro-viral and defensive functions of chaperones. This could be achieved, for example, through CRISPR-Cas-mediated knockdown or mutation of domains in specific HSP isoforms that are preferentially exploited by viruses, while leaving the immunity-related isoforms intact. Identifying such pro-viral isoforms is a key priority. Finally, genome editing using CRISPR/Cas, particularly Cas12b, which has demonstrated optimal efficacy at high temperatures, could be employed to directly edit genes enhancing thermotolerance or to modify chaperone-binding sites on NLR proteins.

## Figures and Tables

**Table 1:** Dichotomous Roles of HSP70 and HSP90 in Plant-Virus Interactions. Abbreviations: VRC: Viral Replication Complex; RdRp: RNA-dependent RNA polymerase; CP: Coat Protein; ETI: Effector-Triggered Immunity; NLR: Nucleotide-binding Leucine-rich repeat (receptor); RISC: RNA-induced silencing complex.

Chaperone	Function Type	Mechanism/Role	Key Interacting Partners	Example Viruses	Source
HSP70	Pro-viral	VRC Assembly: Localization and membrane insertion of replicase proteins; RdRp activation.	Viral replicase proteins (p33/p92); Phospholipids	Tomato bushy stunt virus (TBSV)	[39,40,41]
		VRC Assembly: Prevention of replicase protein aggregation.	Viral replicase protein (p27)	Red clover necrotic mosaic virus (RCNMV)	[42]
		Nuclear Import of viral proteins.	Coat Protein (CP)	Tomato yellow leaf curl virus (TYLCV)	[43]
	Anti-viral	Cooperation with the HSP90 complex to modulate NLR-mediated immunity.	HSP90, SGT1, RAR1	General (e.g., TMV via N protein)	[44,45]
HSP90	Pro-viral	VRC Assembly: Conformational maturation of replicase protein for binding to viral RNA.	Viral replicase protein (p27); Viral RNA element (YRE)	Red clover necrotic mosaic virus (RCNMV)	[42]
	Anti-viral	ETI: Stabilization and maintenance of NLR immune receptors in an activation-competent state.	NLR proteins, SGT1, RAR1	Tobacco mosaic virus (TMV), Potato virus X (PVX)	[46,47]
		RNA Silencing: Loading of small RNAs (siRNA/miRNA) into Argonaute (AGO) proteins to form a functional RISC.	Argonaute (AGO) proteins	General antiviral silencing	[48,49]

**Table 2:** Comparative analysis of outcomes under different stress types. The table summarizes the impact of individual and combined stressors (viral infection and heat stress) on cellular chaperone resources (HSP70/90 pool) and NLR receptor stability. This model illustrates the “competition for resources” hypothesis, explaining the collapse of ETI-immunity under combined stress.

Cellular State	Available HSP70/90 Pool	NLR Receptor Status	Outcome (Susceptibility)
Normal conditions	High (basal)	Stable (HSP90-SGT1-RAR1)	Virus resistance
Viral infection only	Slightly reduced (competition with VRC)	Mostly stable	Resistance maintained
Heat stress only	Severely reduced (diverted to denatured proteins)	Destabilized (undergoing degradation)	N/A (no virus)
Combined stress (Virus + Heat)	Critically reduced (diverted to denatured proteins)	Destabilized and undergoing degradation	ETI collapse, high viral susceptibility

## Data Availability

No new data were created or analyzed in this study. Data sharing is not applicable to this article.
